# Using text mining and sentiment analysis to analyse YouTube Italian videos concerning vaccination

**DOI:** 10.1186/s12889-020-8342-4

**Published:** 2020-02-19

**Authors:** Annamaria Porreca, Francesca Scozzari, Marta Di Nicola

**Affiliations:** 1 Department of Economic Studies, G. d’Annunzio University of Chieti-Pescara, Viale della Pineta 4, Pescara, Italy; 2 Department of Medical Oral Science and Biotechnology, G. d’Annunzio University of Chieti-Pescara Chieti, Via dei Vestini 31, Chieti, Italy

**Keywords:** Vaccinations, YouTube, Sentiment analysis, Pro-vax, No-vax

## Abstract

**Background:**

Recently, social networks have become a popular source of information on health topics. Particularly, in Italy, there is a lively discussion on the web regarding vaccines also because there is low vaccination coverage, vaccines hesitancy, and anti-vaccine movements. For these reasons, in 2017, Institutions have introduced a law to force children to make ten compulsory vaccines for school attendance and proposed a vaccination campaign. On social networks, this law has fostered a fierce discussion between pro-vaccinations and anti-vaccinations people. This paper aims to understand if and how the population’s opinion has changed before the law and after the vaccination campaign using the titles of the videos uploaded on Youtube in these periods.

**Method:**

Using co-occurrence network (CON) and sentiment analysis, we analysed the topics of YouTube Italian videos on vaccines in 2017 and 2018.

**Results:**

The CON confirms that vaccinations were very disapproved before the law. Instead, after the communication campaign, people start to be less critical. The sentiment analysis shows that the intense vaccination campaign also promoted by medical doctors pushed the sentiment to change polarity from a prevailing negative opinion in 2017 (52% negative) to a positive one in 2018 (54% positive).

**Conclusion:**

At the population level, the potential misinformation of social networks could be significant and is a real risk for health. Our study highlights that vaccination campaigns on social networks could be an essential instrument of health policies and a sharp weapon to fight ignorance and misrepresentations of non-qualified people influencing individuals’ decision-making.

## Background

The vaccine coverage provides information on the efficiency of the vaccination system and the trust of the vaccination practice. Currently, Europe faces many challenges, including the spreading antivaccination sentiment [1]. In many countries, there are people refusing vaccinations for themselves and their children, and promoting the idea that immunization is a way to do business. Moreover, there are also other reasons why vaccination practises are not shared by everyone [2–4]. For example, from 2016 onwards, in the USA and Europe, many antivaccination movements were pushed by the “*Vaxxed: From Cover-Up to Catastrophe*”. The latter is an American pseudoscience documentary film directed by Andrew Jeremy Wakefield, who was a British medical supporting the causal relationship between the trivalent MPR vaccine (measles, mumps, rubella) and the appearance of autism and intestinal disease. This theory stimulated people to lose their belief in vaccines, and thus it contributed to lead to the so-called “*vaccine hesitancy phenomenon*” causing a vaccination coverage reduction. The vaccines hesitancy has created health alarmism by pushing countries and international organizations, such as the World Health Organization [5, 6], to look for a way to contain this phenomenon and promote the safety of vaccines. Among the countries of the European Union, in Italy, from 2013 onwards, vaccination coverage has been showing a decreasing trend. Therefore, in 2017, a measles epidemic caused 5000 cases and four deaths [7]. For this reason, on the 7th of June 2017, the Italian Institutions approved the Lorenzin’ decree-law, which increased the number of compulsory vaccinations from four to ten [8]. However, this legislative manoeuvre obtained dissent from a slice of the population divided into two sides: the anti-vaccine people (encompassing who supports freedom of choice in vaccination and thus promotes the non-compulsoriness of vaccinations) and pro-vaccination people who are active in promoting positive campaigns. Both anti-vaccine and pro-vaccine people use the web to share their opinions. Today, many people find information on the World Wide Web (WWW); indeed, recent studies showed that more than half of the population has access to the Internet, and most web users seek health information there (e.g [9, 10]). Moreover, health information is more and more available on the Internet [11, 12]. There are many professional health web sites, app or social network groups to support and inform population but sometimes the quality of health information on the WWW could be a real danger for people [13, 14]. Social networks permit individuals to quickly create, share, and retrieve information, and allow anyone to give opinions and spread their message (also to anti-vaccination activists). Social networks thus contribute to false myths and misinformation about vaccines with a consequent negative impact on people’s willingness to be vaccinated [4, 15].

The aim of the paper is to analyze the online sentiment to understand if the Italian legislative intervention on vaccination is shared by the population and which are the arguments connected with this topic when people share information on YouTube. Indeed, because monitoring social networks could be a good proxy to evaluate vaccine hesitancy, understanding people thinking can guide policy-makers to plan effective information campaigns able to change people mindset regarding vaccinations.

Specifically, we focused on Italy because “*Among the European countries, Italy has one of the highest levels of scepticism related to effectiveness and safety of vaccines*” [16–18]. Moreover, to solve this problem, the Italian Institutions approved the Lorenzin’ decree-law, and thus we are also interested in understanding the effects of this manoeuvre on the sentiment of the population.

According to the “*We are Social - Digital in 2018 Report*”, the internet users in the world exceed four billion, and in Italy, they are more than 43 million; this means that today, more than half of the world’s population is online. In the world, Facebook has much greater penetration than YouTube [19] whereas, in Italy, YouTube dominates the video-based social media platforms. It was on YouTube that the North American anti-vaccine movement was able to upload and share conference recordings free of charge to a wider audience. Compared to the past, when tapes of proceeding had to be ordered by mail, YouTube allowed autism awareness groups to host instant discussions, comments, and information-sharing easily [20–24].

The main contribution of this research to the existing literature on the problem of vaccination hesitancy is to propose the use of text mining and sentiment analysis. Indeed, to educate people increasingly inclined to use WWW as a source of information and to understand their mindset, policy-maker need appropriate tools capable of dealing with the new digital age. Effectively, these instruments could be adopted by public authorities to understand what people’s concerns are about health, what opinion the population has regarding the health policies implemented by the institutions, and finally to know if the policies and campaigns implemented have had the expected results.

This research uses a networked mindset to tackle vaccine hesitancy. Specifically, we aim to understand what the sentiment towards vaccines on YouTube is, and finally to detect if and how the Italians’ opinion has changed from the period before the introduction of the decree-law to the following period, i.e. after the vaccination campaign.

## Methods

We used Netvizz to collect YouTube videos [25]. Netwizz is a tool for extracting data from the YouTube platform via the YouTube API v3 [26]. The search query is “*vaccin*”. The search for the query took place concerning the period from the first of May to the first of October for both the year 2017 and 2018. The iterations used to search the query were chosen equal to ten (fifty items for every iteration) and has been specified to search for each day of the timeframe. The tool provided 3777 videos for 2017 (1898 were adopted for the analysis) and 3788 for 2018 (822 were used for the analysis). The exclusion criteria were a language different from the Italian one; titles not containing the subject under investigation; and finally videos with ambiguous titles were not taken into account.

We highlight that Netwizz also provided the following variables about videos: number of dislikes and likes, duration, comment count, and view count. We used Google Trends to determine the period in which to focus the search of the query and which was the sought-after query in terms of vaccines.

We considered the maximum and minimum of the query’s functions of each year to capture the time-variability of the queries before and after the vaccination campaign. This criterion led to identifying 6 months in 2017 (from May to October) while, for 2018, a two-months range (from June to July). To guarantee the comparability of the two periods and considering that the June–July period lies within the May–October interval, we refer to the May–October periods both in 2017 and 2018.

All relevant material for Italians is available only with the local language; in truth, even on the website of the Ministry of Health in Italy, it is quite difficult to find material in a language other than Italian! Indeed, todate, regarding vaccinations, the English version is still under construction [27]. Therefore, the choice of the query was quite trivial because we compared the query’s functions on Google Trends for the following words: “*vaccino*” (in English “*vaccine*”), “*vaccini*” (in English “*vaccines*”), “*vaccinazioni*” (in English “*vaccinations*”), “*morbillo*” (in English “*measles*”), and “*autismo vaccini*” (in English “*autism vaccines*”). Because both “*vaccino*” and “*vaccini*” were much more searched on the web than the others, we used the query “*vaccin*” that allowed us to search for both the singular and the plural in the Italian language [28].”

To understand the main topic about vaccines on YouTube, we analyzed the data by using the co-occurrence network (CON) based on Freeman’s betweenness centrality Index by KH Coder 3.0 [29, 30]. Betweenness centrality is the measure of the degree to which one node is “between” other nodes in a network; this means that this node could act as mediators on the network, regardless of the frequency of connectivity [29]. After the user fixes some coding rules, CON takes into account co-occurrence relations among terms. The degree of co-occurrence was determined using the Jaccard similarity coefficient [31]. The value of the latter index is a scalar and ranges from 0, indicating no spatial overlap between two sets of binary segmentation results, to 1, indicating complete overlap. To conduct the CON, we used lexicon coding rules to exclude logic operators such as “*e*”, “*o*”, “*non*”, demonstrative adjective, people names, other language words and meaningless words. To analyze the sentiment during the considered periods about the content of the co-occurrence networks, we used the function “*get_nrc_sentiment*” based on the NRC sentiment dictionary to calculate the presence of eight different emotions and their corresponding valence in a text file from the “*syuzhet*” R environment package [32]. The words in each sentence are compared to a dictionary and tags positive or negative words by + 1 or − 1, respectively. Before testing differences into emotions between the two periods, we rescaled the sentiment vectors to the normalized axis, and we got re-sampling vectors for comparing them. T-tests were performed to test differences in the mean between the year’s sentiment and video characteristics: duration (min), view count, number of likes, number of dislike and comment count. Statistical significance was set at *p* < 0.05. For each variable in the study, we have included descriptive statistics.

## Results

Table 1 shows the descriptive statistics of the YouTube videos in Italian language containing a vaccination theme in the year 2017 and 2018. On average, the duration of the videos and the number of views decrease from 2017 to 2018. It is interesting to note that the number of likes, on average, is higher in 2018 than in 2017. The same is for the number of comments, and thus 2018 videos have reached greater popularity.

**Table 1:** Descriptive statistics of YouTube videos about vaccines in the Italian language in 2017 and 2018, respectively and *p*-value of t.test comparisons

*Variable*	*2017 (N = 1898)*		*2018 (N = 822)*		*p-value*
	*Number of videos*	*Mean ± SD*	*Number of videos*	*Mean ± SD*	
Duration (min)	1885	503.40 ± 693.70	811	404.66 ± 586.18	0.278
View count	1891	2621.68 ± 19,243.40	820	1599.40 ± 9233.13	0.633
Number of Like	1799	69.24 ± 622.43	787	85.27 ± 598.93	0.853
Number of Dislike	1799	7.21 ± 31.38	786	6.21 ± 37.04	0.837
Comment count	1720	17.32 ± 80.89	822	18.41 ± 106.30	0.935

**Fig. 1:**
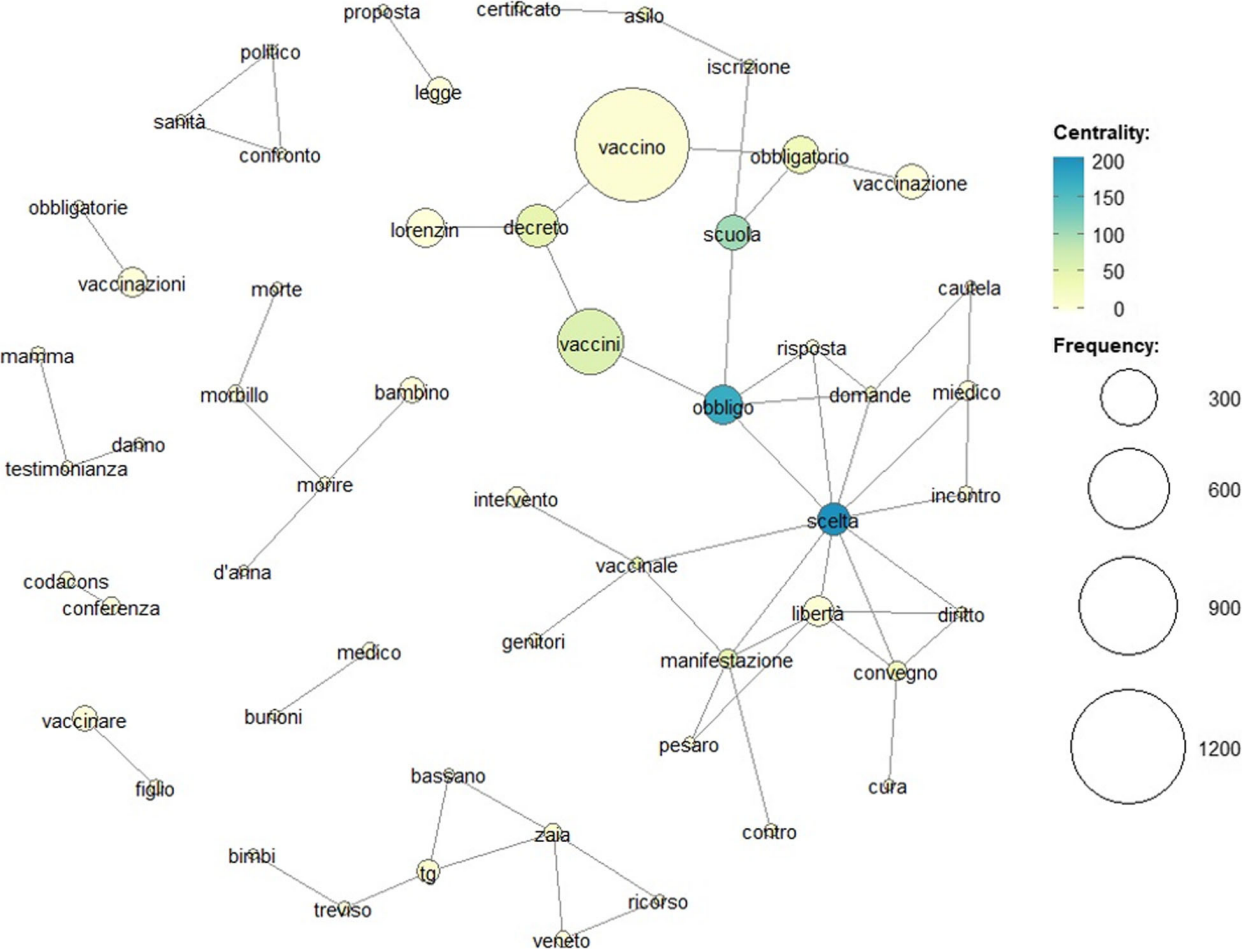
Co-occurrence Network (CON) based on Betweenness centrality index for Italian Youtube videos concerning vaccines in 2017 (from May to October). Nodes corresponding text in english is: “zaia” = Zaia (italian politician), “veneto” = Veneto (italian region), “vaccino = vaccine, “vaccinazione, vaccinale” = vaccinations, “treviso” = Treviso (Italian city), “tg” = newscast, “testimonianza” = evidence, “scuola” = school, “scelta” = choice, “sanità” = “risposta” = answer, “ricorso” = contestation, “proposta” = motion, “politico” = politician, “pesaro” = Pesaro (italian city where took place no-vax parade), “obbligo, obbligatorio” = compulsory, “morte” = death, “morire” = die, “morbillo” = measles, “miedico” = Miedico, “manifestazione” = manifestation, “libertà” = freedom, “iscrizione” = enrolment, “intervento” = intervention, “incontro” = meeting, “genitori” = parents, “figlio” = son, “domande” = questions, “diritto” = right, “danno” = damage, “d’anna” = D’anna (senator against vaccines), “cura” = care, “contro “= against, “confronto” = confrontation, “conferenza” = conference, “codacons” = Codacons (italian non-profit association for the protection of consumers and the environment), “certificato” = certified, “cautela” = prudence, “burioni” = Burioni (Italian medical doctor pro-vax), “bimbi” = kids, “bassano” = Bassano (italian city), “asilo” = kindergarten

**Fig. 2:**
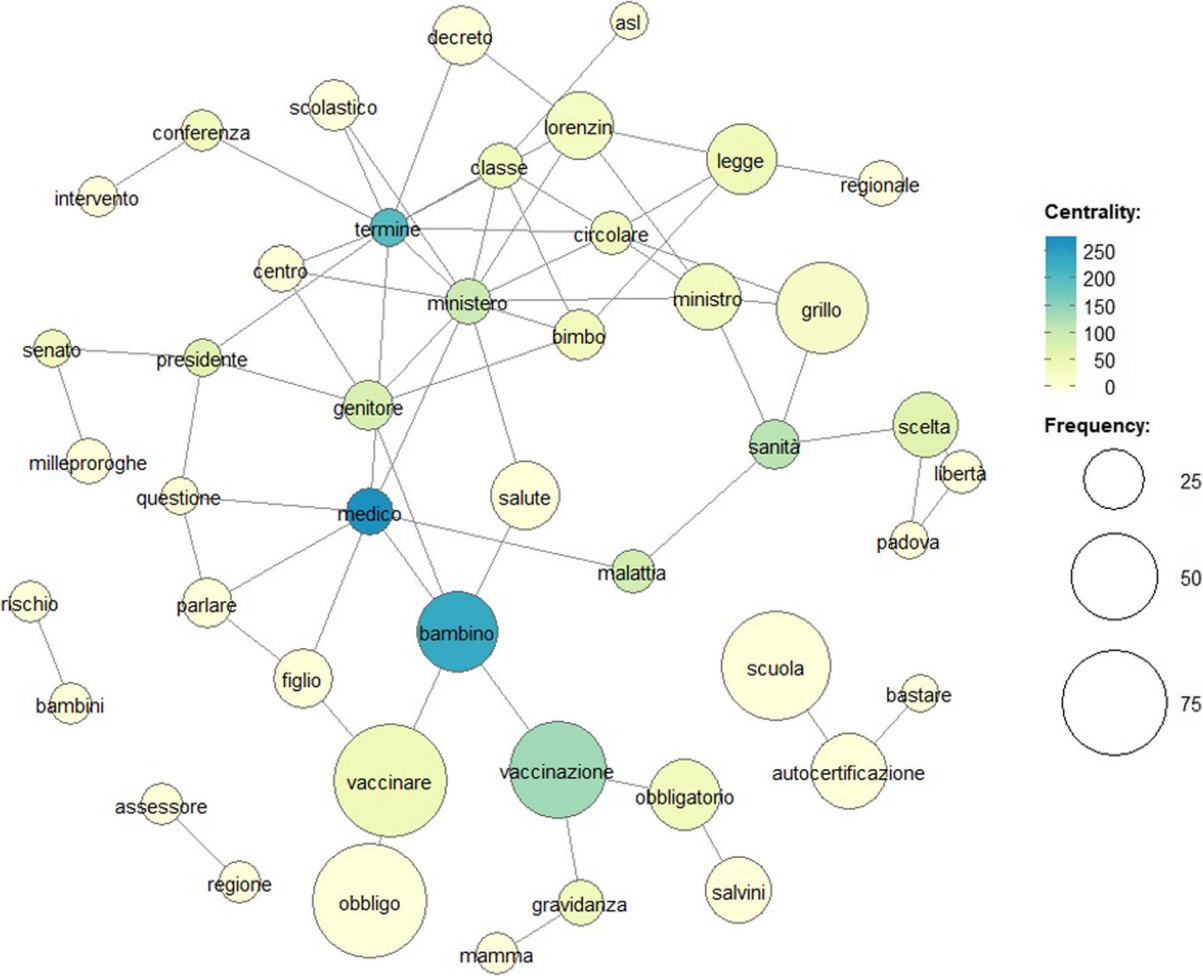
Co-occurrence Network (CON) based on Betweeness centrality index for Italian Youtube videos concerning vaccines in 2018 (from May to October). Nodes corresponding text in english is: “conferenze” = conference, “scolastico,scuola” = school, “decreto” = decree, “classe” = class, “ministero” = ministry, “intervento” = intervention, “centro” = center, “termine” = deadline, “circolare” = school memorandum, “bimbo,bambino” = child, “bambini” = children, “obbligatorio, obbligo” = compulsory, “mamma” = mother, “vaccinare” = vaccine, “figlio” = son, “rischio” = risk, “regione” = region, “assessore” = councillor, “autocertificazione” = self-declaration, “bastare” = enough, “vaccinazione” = vaccination, “gravidanza” = pregnancy, “parlare” = talk about, “questione” = issue, “senato” = senate, “presidente” = president, “genitore” = parent, “salute, sanità” = health, “malattia” = illness, “scelta” = choice, “libertà” = freedom, “ministro” = minister, “regionale” = regional, “legge” = law, “medico” = medical doctor, “asl” = local health authority, “salvini” = Salvini (italian politician), “grillo” = “Giulia Grillo (italian health ministry from 1 June 2018)”, “lorenzin” = Lorenzin (italian politician responsible for the decree for increased compulsory vaccinations; up to 1 June 2018 italian health ministry), “padova” = Padua (italian city where took place many protests no-vax, no-vaccination compulsory)

Figures 1 and 2 show the textual analysis through the CON in 2017 and 2018, respectively. Figure 1 shows that, in 2017, the terms “*compulsory*” and “*choice*” have high degrees of centrality within the network. But also the terms “*school*” and “*decree*” have a high centrality within the network although their frequency is low. Particularly interesting is the fact that the term “*choice*” is connected and many other terms pertaining to the subject such as “*vaccination*”, “*freedom*”, “*law*”, “*meeting*”, “*doctor*”, “*questions*”, “*compulsory*”, “*manifestation*”. Moreover, we observe many connected cliques and small clusters stressing strong concern and perplexities. In summary, the graph underlines that there is an interesting connection between vaccinations and freedom of choice.

Figure 2 shows that, in 2018, the most central words in the network in order of importance are: “*medical doctor*”, “*child*”, “*deadline*”, “*vaccination*”, “*ministry*”, “*health*”, and “*parent*”. The betweenness centrality index of these words does not coincide with the frequency of appearance in the text, this means that although they do not appear very frequently in the speech, they connect most of the themes acting as bridges to reach the most isolated nodes. The word “*ministry*” is also the node a high betweenness centrality being the one most connected in the network having a high degree centrality. It can be noticed how taking as a “*ministry*” centre this constitutes a centre for the formation of a connected cluster that includes topics concerning the Lorenzin decree, conferences, health, regulation at the regional and scholastic level. Interesting is the change in the importance of terms such as “*freedom*”, “*choice*”, “*risk*”, and “compulsory”; hence, it suggests that the previous negative discussions about vaccinations are weakened, bringing the speech back to less polemical tones.

**Fig. 3:**
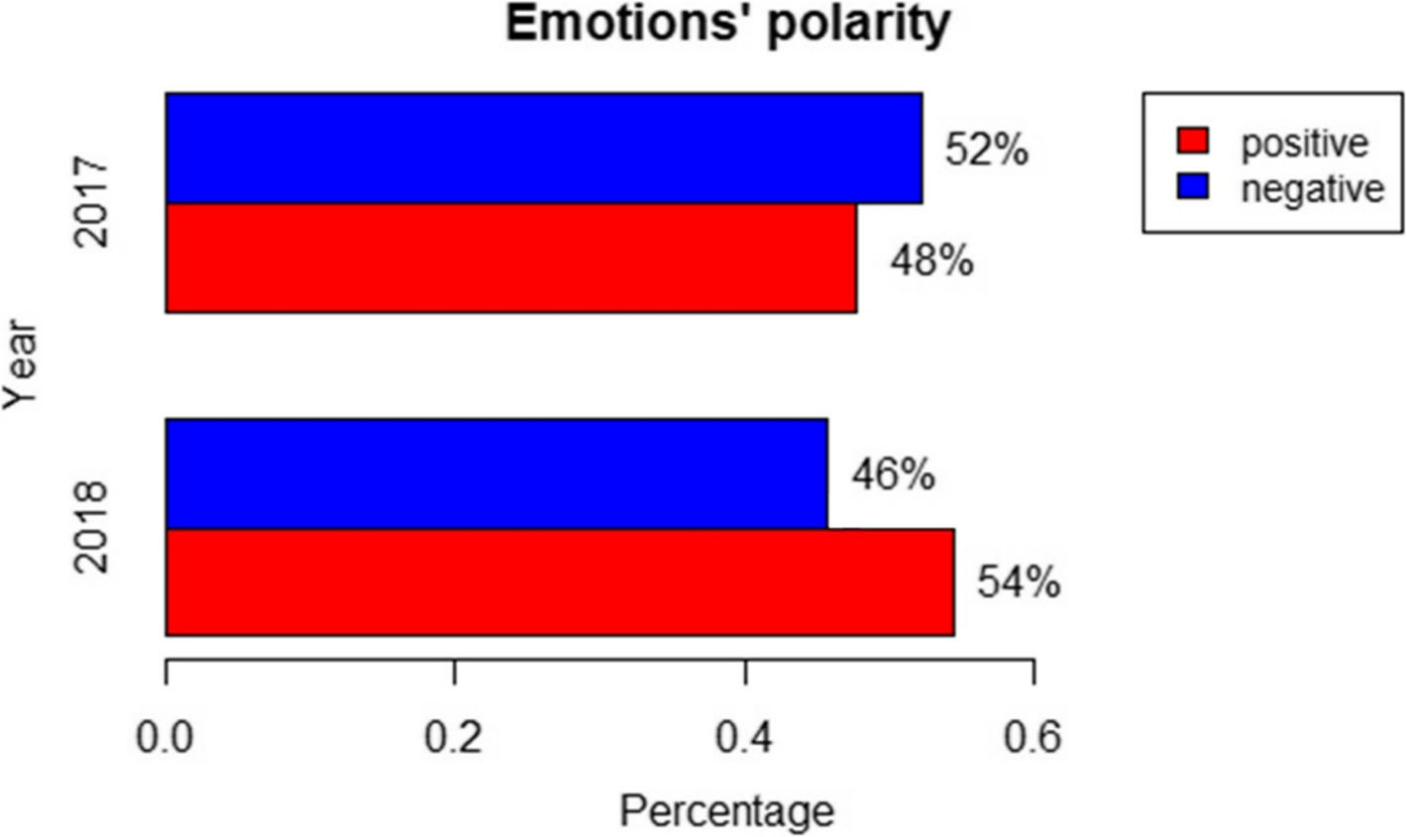
Emotions’ polarity in 2017 and 2018. F-Test equal to 0.016. ANOVA computed using the sentiment scores

Figure 3 shows the barplot of the emotions’ polarity in 2017 and 2018. In 2017, the prevailing sentiment was the negative one (52%), whereas, in 2018, the positive sentiment was 54%.

**Fig. 4:**
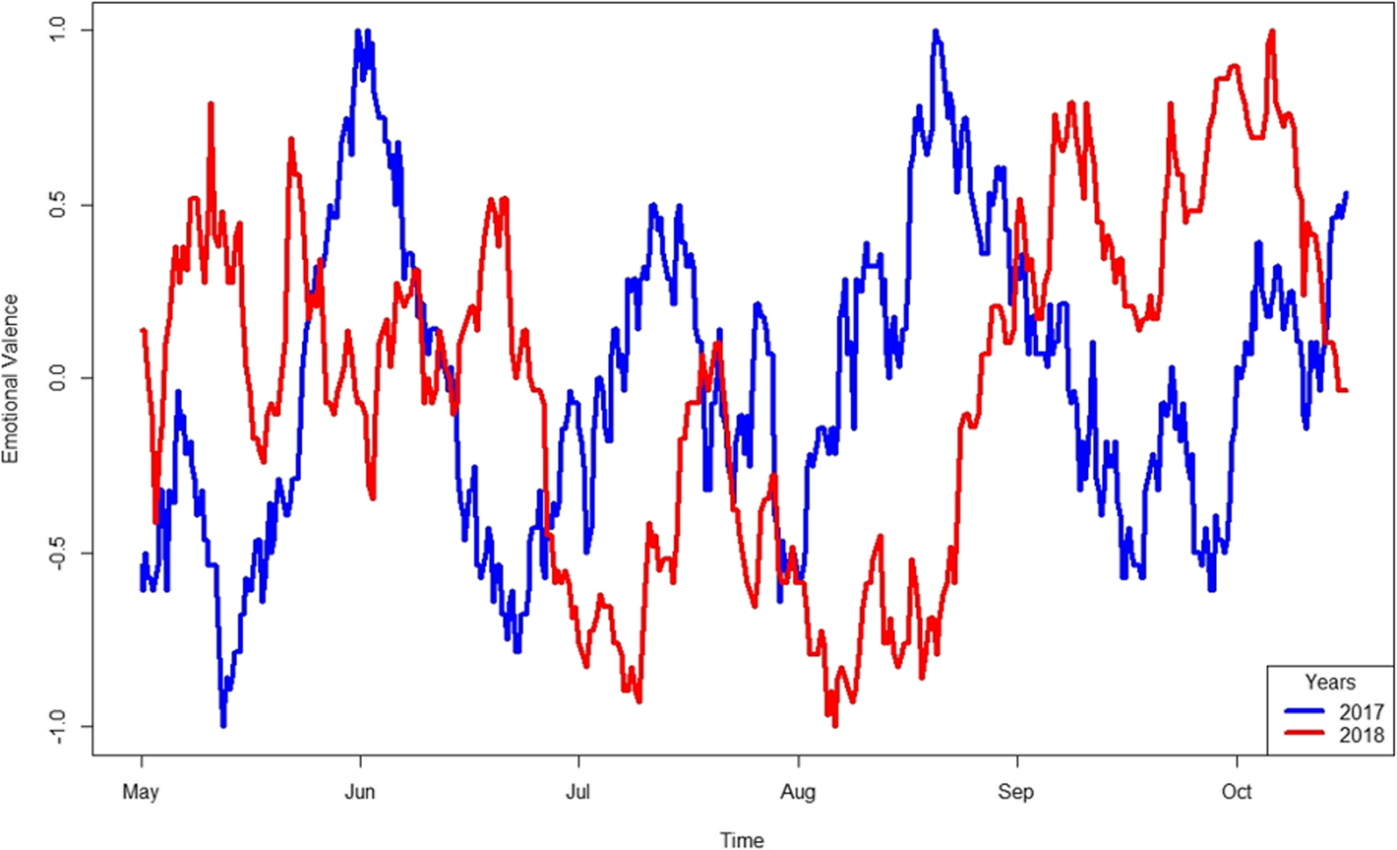
Sentiment about vaccines in Italian Youtube videos titles in 2017 and 2018

Figure 4 shows sentiments’ change over time from 2017 (mean = 0.46 and SD = 0.29) to 2018 (mean = 0.53 and SD = 0.30) assessed in the different sixth-months periods. T-test confirms a statistically significant change (*p* = 0.02).

## Discussion

In Italy, the vaccination coverage strongly increased from the 31st of December 2017 to the first 6 months of 2018 [7]. Indeed, the minimum threshold (95% vaccination coverage) recommended by the WHO was reached [6]. This result can be extrapolated by a medium-term survey carried out to assess the impact of the law on compulsory vaccinations in the cohorts of children born in 2015, 2014 and 2010 [7]. The Lorenzin’s decree has undoubtedly pushed up the number of compulsory vaccines; however, it would be interesting to understand if the communication campaigns and commitment by the experts on the web had some effects on the opinion regarding the vaccination practice and their possible risks. In recent years, as Italian National Institute of Statistics (ISTAT) official data confirm, the number of Internet users in Italy has increased [33–35]. In 2017, the Italians represented 7.8% of European internet users [36]. Due to the growth in the number of people who use the WWW to look for health-related information, public health policy-makers should consider the consequences of such use at the population level. For this reason, the analysis of web search trends and social network data could represent an exciting proxy for vaccine hesitancy [37, 38]. In 2017, YouTube was identified as the most adopted social network in Italy [6]. Nevertheless, few studies have analysed how people exploit this social network for health purposes and to make decisions on their future behaviour. Indeed, these platforms and their contents have the potential to influence people health behaviours [20, 39], and thus the influence of vaccine-critical content and increase in using these platforms to share health information is a cause for concern for public health authorities. As also stressed by MacDonald, poor or inappropriate communications can lower the vaccination coverage and contribute to the hesitation of vaccination [40]. However, the quality of vaccine-related material available online is varied, and internet search engines often bring people to anti-vaccine or low-quality web information; therefore, the potential for misinformation is relevant [41, 42]. Generally, who use a search engine is likely to focus on the potential risks of the vaccines and thus find anti-vaccination websites. Therefore, widely spread bad contents may contribute to reduce vaccine uptakes and increase the risk of preventable diseases. Hence, at the national level, better communication in the field of vaccination is desirable to counteract the phenomenon of vaccine hesitancy, including the development of digital tools to facilitate progress towards empowerment and changes in citizens’ behaviour. For these reasons, it is vital to stimulate communication campaigns to encourage people healthy lifestyles and develop materials for health education. In fact, communication plays a crucial role in vaccination hesitancy, and thus it is necessary to identify the main communication channels to use resources and strategies that can influence public opinion [43]. To the best of our knowledge, this is the first study that tries to assess the contents of YouTube videos and their associated sentiment on compulsory vaccination using text mining and sentiment analysis tools. This paper shows that, after the vaccination campaigns and promotion, and also after health ministry change (from 1 June 2018 Giulia Grillo is the Italian health ministry), the topics and sentiment about vaccines on YouTube have changed from 2017 to 2018 (see also Figs. 1 and 2). The vaccines arguments in 2017 on YouTube in Italy were very criticises about their obligatoriness. Most titles expressed dissents and mostly negative sentiment in the linked comments. In 2018, the arguments became less harsh towards vaccination and Lorenzin’s decree, and there was even an inversion of polarity in the sentiment.

The final aim of this paper is to stimulate public authorities to invest in the digital communication for spreading health information, raising awareness among increasingly younger users, and reaching a type of communication that is increasingly popular. Indeed, this kind of information is essential to educate people increasingly inclined to use WWW as a source of information. The competent authorities should invest and apply policies for correct information on the web by financing projects and qualified operators capable of coping with the new digital age.

### Limitations

Although a considerable number of videos were selected for analysis, availability and access to API, and time limitations made it unfeasible to examine all videos. For these reasons, we have conducted an analysis completely excluding human judgment. Despite these limitations, to the best of our knowledge, this is the first study to examine YouTube vaccination’s contents in Italy using a text mining and sentiment analysis procedure not affected by a human classification which implies an intense subjectivity in the attribution of sentiment scores such as to cause a strong bias in the study.

## Conclusions

All European countries have vaccination policies and, in particular, Italy is the first European country to approach comprehensively the growing vaccine hesitancy. Following the disinformation about vaccines, vaccination coverage in Italy has fallen below the critical threshold of 95% for various diseases. This situation can have serious consequences, both for the health of unvaccinated children and for those around them. The issue of compulsory vaccinations introduced by the Lorenzin’s decree has aroused much discussion within the WWW. Social networks express opinions from both experienced and non-experts and reflect the feeling related to the subject of vaccination in Italy. Although in 2017 vaccinations have been highly criticized regarding their safety and ethics, and thus recorded a negative opinion by the Italian YouTube users, in 2018, there is an inversion of polarity in the general sentiment. Indeed, in 2018, users discussed this topic less critically, no longer emphasising the risk and danger of the vaccine but trying to understand and address the consequences of the law-decree regarding the information of the documentation required for access to school.

The ongoing debate about the adoption of mandatory vaccination policies, especially in the light of the fragmentation of the vaccination regulatory framework still challenges the scientific community, the political stakeholders and the general population in Italy. Because the pro-vaccine campaigns are designed to raise awareness among parents about the importance of vaccinating their children, policymakers should adopt the appropriate campaigns according to the society they live in. Lawmakers and public health authorities should play a fundamental role in informing the public and should use the media to do this so people can make informed choices. But politics are primarily influenced by what appears in ‘old’ media, such as television, radio and newspapers, and ‘new’ media such as social networks and the Internet are not exploited as necessary. Besides, the link between the media and politics can have a notable influence on vaccination coverage, and thus policymakers should use the ‘old’ and ‘new’ media for enhancing health-related information that can strengthen vaccination coverage.

This study shows how the public authority must take into account the WWW and its potential dangers, adopting communication strategies to manage as much as possible the information from social networks by contrasting fake news.

## Data Availability

Data are available on Netwizz tool (https://tools.digitalmethods.net/netvizz/youtube/) setting it on five iterations of data and italian language, using as research query “vaccin” .

## Abbreviations

CON: Co-occurrence network
ISTAT: Italian National Institute of Statistics
WHO: World Health Organization
WWW: World Wide Web
